# Effects of Grape Pomace Polyphenolic Extract (Taurisolo^®^) in Reducing TMAO Serum Levels in Humans: Preliminary Results from a Randomized, Placebo-Controlled, Cross-Over Study

**DOI:** 10.3390/nu11010139

**Published:** 2019-01-10

**Authors:** Giuseppe Annunziata, Maria Maisto, Connie Schisano, Roberto Ciampaglia, Viviana Narciso, Gian Carlo Tenore, Ettore Novellino

**Affiliations:** Department of Pharmacy, University of Naples “Federico II”, Via Domenico Montesano 49, 80131 Naples, Italy; maria.maisto@unina.it (M.M.); connie.schisano@unina.it (C.S.); roberto.ciampaglia@unina.it (R.C.); viviana.narciso@gmail.com (V.N.); giancarlo.tenore@unina.it (G.C.T.); ettore.novellino@unina.it (E.N.)

**Keywords:** TMAO, grape marc, polyphenols, resveratrol, nutraceutical

## Abstract

Trimethylamine N-oxide (TMAO) is considered a novel risk factor for cardiovascular diseases. Several studies demonstrated that polyphenols are able to inhibit the growth of TMA-producing bacterial strains, and resveratrol (RSV) reduced TMAO levels in mice. In the present study, we evaluated the TMAO-reducing effect of a novel nutraceutical formulation containing grape pomace extract in humans (Taurisolo^®^). The Taurisolo^®^ polyphenol content was evaluated by a High Performance Liquid Chromatography-diode-array detector (HPLC-DAD) method, and RSV was monitored as an indicative marker. After in vitro GI digestion, intestinal bioaccessibility of RSV was 92.3%. A randomized, placebo-controlled, cross-over trial was carried out to evaluate the TMAO-reducing effect of Taurisolo^®^. In acute, the maximum levels of RSV were detected both in serum and whole blood 60 min after the administration of Taurisolo^®^; in chronic, a significant increase of RSV was detected in serum after the 4-week treatment. After 4 weeks, the levels of TMAO were significantly decreased in the treatment group compared to placebo (63.6% vs. 0.54%, respectively, *P* < 0.0001). In conclusion, our data show that Taurisolo^®^ may represent a novel and useful natural remedy to reduce prognostic markers for incident cardiovascular events. Undoubtedly, further in vitro and in vivo studies need to be performed in order to elucidate possible mechanisms of action and corroborate our preliminary results.

## 1. Introduction

Trimethylamine N-oxide (TMAO) is currently recognized to be a novel risk factor for cardiovascular diseases (CVD) [1], including stroke [2,3,4,5] and other major adverse cardiovascular events [6,7,8,9,10,11,12], heart failure [13] and atherosclerosis [14]. TMAO is an amine oxide with structural formula (CH_3_)_3_NO [1,15] primarily deriving from choline and l-carnitine metabolized by the gut microbiota [1], mainly by *Clostridia*, *Shigella*, *Proteus*, *and Aerobacter* [16], producing trimethylamine (TMA). The complex metabolism of TMAO has been extensively described. In particular, after its production in the gut, TMA is absorbed and travels to the liver, where the flavin-containing monooxygenase-3 (FMO3) is responsible for its oxidization [7,8,17]. About half of the circulating TMAO is excreted through urine, breath, and sweat [18,19,20], while the remaining part undergoes further microbiota metabolism to TMA [21,22]. Interestingly, several foods containing high amounts of choline, l-carnitine, and lecithin, such as eggs, red meat, salt-water fish, seafood, and dairy products, have been considered dietary sources of TMAO [8,23].

In this context, thus, the microbiota remodeling (including the use of prebiotic and probiotic supplements) and dietary interventions (including diets rich in fiber and antioxidants) appear to be as novel and useful approaches for the management of high TMAO levels-related diseases [24].

Besides the well-known antioxidant activity, evidence showed that polyphenols are also able to act as bactericide and bacteriostatic agents against some specific bacterial strains, mainly *Clostridia* [25], suggesting that diets rich in polyphenols or supplements may represent a strategy for microbiota remodeling. In addition, an animal-based study demonstrated the ability of resveratrol (RSV) in reducing TMAO levels in mice [26]. Furthermore, RSV is effective in remodeling the microbiota composition by (i) increasing *Lactobacillus* and *Bifidobacterium* growth [26], (ii) increasing the *Bacteroidetes/Firmicutes* ratio and (iii) reducing the *Enterococcus faecalis* growth [27].

Based on this evidence, the present study aimed to demonstrate the TMAO-reducing effect of grape pomace, an agri-food by-product rich in polyphenols. Particularly, a randomized, placebo-controlled, cross-over clinical trial was conducted on healthy subjects evaluating the efficacy of a novel nutraceutical formulation containing grape pomace polyphenolic extract microencapsulated with maltodextrins, registered as Taurisolo^®^, in acid-resistant capsules.

## 2. Materials and Methods

### 2.1. Reagents

All chemicals, standards, and reagents used were either analytical and high-performance liquid chromatography (HPLC)-grade reagents. The water was treated in a Milli-Q water purification system (Millipore, Bedford, MA, USA) before use. Chemicals and reagents used to simulate the gastrointestinal digestion: potassium thiocyanate (KSCN), potassium chloride (KCl), sulfate (Na_2_SO_4_), monosodium phosphate (NaH_2_PO_4_), sodium bicarbonate (NaHCO_3_), hydrochloric acid (HCl), and sodium chloride (NaCl). The enzymes used were α-amylase, pepsin (≥250 U/mg solid) from porcine gastric mucosa, and pancreatin (4 × USP) from porcine pancreas and were purchased from Sigma-Aldrich (Milan, Italy).

### 2.2. Grape Pomace Extract Supplement Preparation

Taurisolo^®^ was obtained from Aglianico cultivar grape, collected during the harvest in autumn 2016. The supplements used in this study consisted of gastro-resistant capsules containing grape pomace polyphenolic extract microencapsulated with maltodextrins (300 mg/cps). The extract was encapsulated in gastro-resistant capsules due to our previous knowledge of salivary and gastric digestion of some polyphenolic components. The product was formulated by the Department of Pharmacy, University of Naples “Federico II” (Naples, Italy). Large scale production of Taurisolo^®^ has been accomplished by MBMed Company (Turin, Italy). Grape has been extracted with pure ethanol, and the obtained extract solution has been kept at −20 °C for 24 h to allow sugar elimination. After centrifugation, the extract underwent a spray-drying process with maltodextrins as support, obtaining a fine powder, which has been used to formulate gastro-resistant capsules.

### 2.3. In Vitro Gastrointestinal (GI) Digestion

In order to evaluate the bioaccessibility of RSV, in vitro GI digestion protocols were performed on two versions of the nutraceutical formulation (acid-resistant (AR) and non acid-resistant (NAR) capsules), following the procedure described in our previous study [28]. The procedure used is described in details in the Supplementary Materials.

### 2.4. HPLC/DAD Analyses of Taurisolo^®^ Polyphenols

The main polyphenols contained in Taurisolo^®^ were monitored by HPLC-diode-array detector (DAD) analysis, following the method described by Giusti et al. [29] and Silva et al. [30]. Information about the HPLC-DAD system used and method conditions are reported in the Supplementary Materials.

### 2.5. TMAO Quantification

The quantification of TMAO serum levels was performed according to the high-performance liquid chromatography-mass spectrometry (HPLC/MS) method described by Beale and Airs [31] and reported in our previous studies [32,33], with slight modifications. Briefly, 160 µL of methanol was added to 80 µL of plasma, in order to precipitate serum proteins. The mixture was vortex-mixed for 2 min, centrifuged at 12,000 rpm for 10 min (4 °C) [32,33,34], and the supernatants were used for the HPLC-MS analysis. The HPLC/MS system used and method conditions are reported in the Supplementary Materials.

### 2.6. Study Design, Setting and Population

Healthy subjects, aged 25–35 years were enrolled in July 2018 from personnel and students of our Department. All subjects gave their informed consent for inclusion before they participated in the study. The study was conducted in accordance with the Declaration of Helsinki, and the protocol was approved by the Ethics Committee of AO Rummo Hospital (Benevento, Italy) (protocol n. 123512 of 18 June 2018).

This was a 70-day randomized, double-blind, single-center, placebo-controlled, crossover trial. The subjects were randomly divided into two groups. They were followed by a crossover design consisted of run-in period (7 days), washout period (7 days), and intervention period (28 days). During the intervention period, each subject was given 300 mg Taurisolo^®^ in acid-resistant capsules or a placebo (identical capsules containing only maltodextrin) twice daily, accompanying their two main meals. Subjects were instructed to annotate their dietary habits in a food diary and to maintain their habitual physical activity patterns for the entire duration of the study. In order to verify the compliance and increase protocol adherence, qualified personnel performed standardized and periodic telephone interviews, reminding patients to complete their daily reports, as previously described. During clinic visits, self-administered questionnaires on quality of life aspects were completed by each patient, and diaries were checked for data completeness and quality of documentation to ensure patient comprehension of the diary items.

Blood samples were collected after 12 h of fasting at days 8, 35, 42, and 70 in 10-mL EDTA-coated tubes (Becton–Dickinson, Plymouth, UK) and plasma was isolated by centrifugation (20 min, 2200 rpm, 4 °C). All samples were stored at −80 °C until analysis. Subjects were instructed to abstain from alcohol consumption and practice of hard physical activity 48 h prior to blood sampling.

Exclusion criteria, randomization, concealment, blinding, data collection, evaluation of nutraceutical treatment safety and statics are detailed in the Supplementary Materials.

## 3. Results

### 3.1. Taurisolo^®^ Composition

The analysis of the main polyphenols contained in Taurisolo^®^ was performed by the HPLC-DAD method. Mean values, expressed in µg/g, are reported in Table 1. Among these, RSV was considered the most representative Taurisolo^®^ polyphenol; for this reason, RSV levels were monitored in all analyses, including bioaccessibility (levels of RSV in the duodenal stage after in vitro digestion) and bioavailability (RSV levels in serum/blood) studies. In addition, serum levels of RSV at the end of the clinical trial were considered as assumption-marker.

### 3.2. Bioaccessibility and Plasma Levels of Resveratrol in Taurisolo^®^

**Figure 1:**
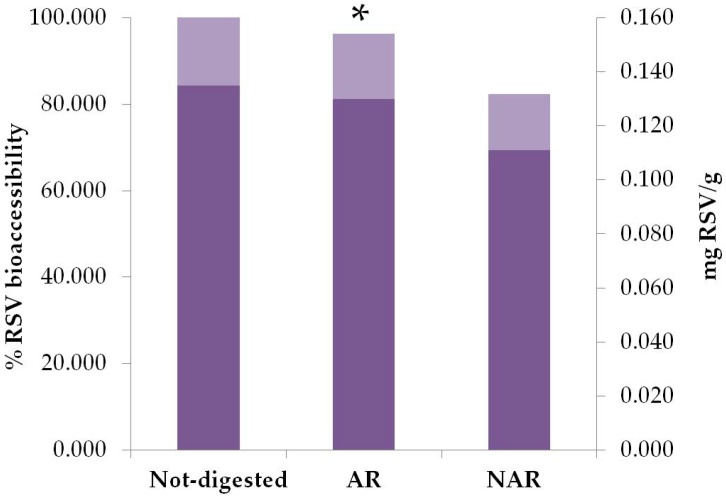
Intestinal bioaccessibility of RSV contained in Taurisolo^®^ after in vitro gastrointestinal digestion on AR and NAR capsules. Data are expressed as mean values in mg RSV per g of extract, and percentage of RSV intestinal bioaccessibility. Statistic significance is calculated by Student’s *t*-test: * *P* = 0.0002, AR bioaccessibility vs. NAR bioaccessibility. RSV, Resveratrol; AR, Acid-resistant capsule; NAR, Non acid-resistant capsule.

In order to evaluate the pharmacokinetics of the Taurisolo^®^ polyphenols, bioaccessibility and bioavailability studies were performed, monitoring RSV as an indicative marker. Bioaccessibility of RSV was evaluated through an in vitro GI digestion performed on AR and NAR capsules containing 300 mg Taurisolo^®^. With AR capsules the intestinal bioaccessibility of RSV was 96.3%, whereas with NAR capsules it was 83% (Figure 1).

For the evaluation of the RSV bioavailability, acute and chronic studies were carried out. In particular, for the acute study, the first day of the clinical trial two AR capsules containing 300 mg Taurisolo^®^ were administrated to each fasting participant and blood samples were collected before (time: 0 min) and after the administration, at different times (30, 60, 120, and 240 min). RSV levels were quantified both in serum and whole blood samples. As shown in Table 2, 60 min after the oral administration of AR capsule containing Taurisolo^®^, the maximum levels of RSV were detected both in serum and whole blood (49.0 ± 0.55 and 14.2 ± 0.40 ng/mL, respectively).

**Figure 2:**
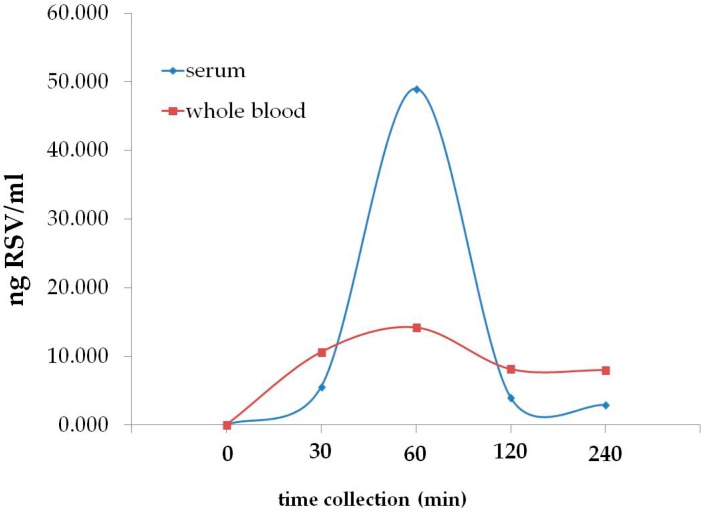
Bioavailability of RSV in acute. RSV levels evaluated in serum and whole blood samples before (time 0) and after (30, 60, 120, and 240 min) the administration of 2 AR capsules containing 300 mg Taurisolo^®^. In both serum and whole blood, the maximum levels of RSV (49.0 ± 0.55 and 14.2 ± 0.40 ng/mL, respectively) were detected 60 min after the nutraceutical administration.

Before and after this time collection, lower levels of RSV were detected in both serum and whole blood, resulting in a typical bell shape, as shown in Figure 2. For the chronic study, RSV amounts were also quantified at the end of the 4-week treatment for each fasting participant. As reported in Table 2, at time ‘0 min’ RSV content was not detected, whereas after the 4-week treatment a mean value of 7.50 ± 0.04 ng/mL was detected in serum samples.

### 3.3. Enrolment and Subject Attrition

**Figure 3:**
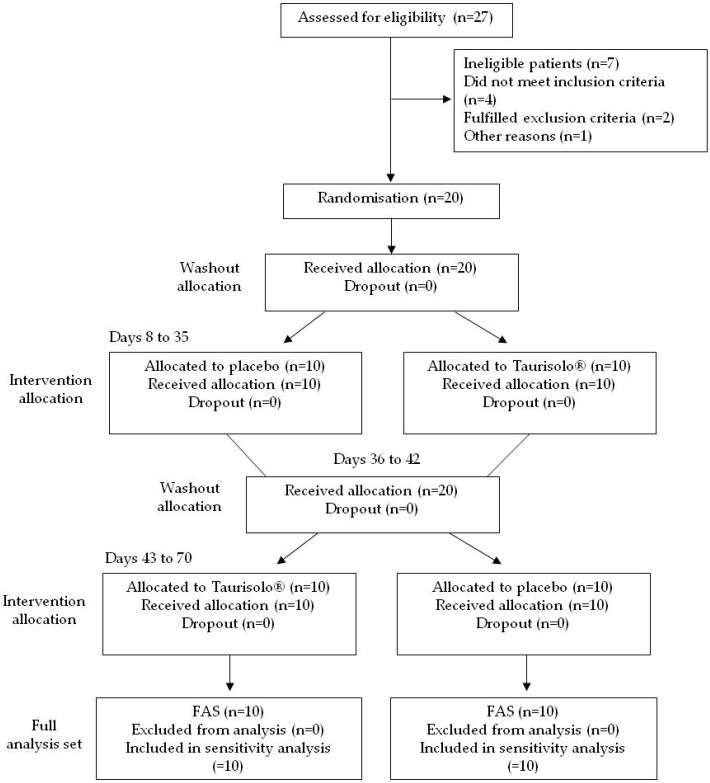
Study flowchart. Study flowchart, according to the consolidated standards of reporting trials (CONSORT). The diagram shows enrolment and primary efficacy endpoints based on patients’ diaries, from prescreening to data collection; and the extent of exclusions, loss to follow-up, and completeness of. Diary documentation available across the entire trial period. FAS, Full analysis set.

Study participants were enrolled in July 2018. A total of 27 subjects were screened for eligibility; 20 were randomized, while 7 (25.9%) did not pass the screening stage. The most common reasons were as follows: four subjects did not meet the inclusion criteria at baseline, two subjects did not fulfill exclusion criteria and one subject refused to participate for no specific reasons. Selected patients were equally divided into two subgroups. Each subject underwent a 7-day washout period before the 28-day intervention period, according to a crossover plan. All participants completed the study. Figure 3 is the flow chart of the study according to the CONSORT PRO reporting guideline [35].

### 3.4. Effect of Taurisolo^®^ on TMAO Levels

Table 3 shows that TMAO serum levels were significantly decreased (Δ% = −63.6%) in the Taurisolo^®^ period compared with placebo period (*P* < 0.0001).

## 4. Discussion

In the present study, we demonstrated the ability of a novel nutraceutical formulation consisting of AR capsules containing Taurisolo^®^ in reducing TMAO serum levels in healthy adults. Particularly, a randomized, placebo-controlled, cross-over study was conducted on 20 healthy subjects. Interventions consisted of AR capsules containing 300 mg Taurisolo^®^ twice daily and equal amounts of maltodextrins as a placebo. After 4-weeks treatment, in all subjects TMAO levels were significantly decreased (mean decrease percentage −63.6%; *P* < 0.0001, compared to placebo). As the study population consisted of healthy adults, TMAO serum levels were expected to fall within the physiological range. Nevertheless, a specific aim of the present study was to validate the efficacy of Taurisolo^®^ in terms of reduction of TMAO serum levels. We observed the ability of Taurisolo^®^ to reduce TMAO blood concentrations, suggesting that it may serve not only as a novel therapeutic strategy for high-risk subjects, but also as a preventive approach, contributing to maintaining values within the normal ranges.

The decrease of the TMAO serum levels is a novel frontier in the reduction of CVD risk. TMAO, indeed, is currently considered to be a novel risk factor for CVD and an oxidative stress biomarker [1]. Normal TMAO blood levels ranged 0.5–5 µM [7,15] and increased TMAO serum levels are associated with a greater CVD risk [1]. Interestingly, higher TMAO urinary [36] and serum [32] levels were correlated with lower Mediterranean diet (MD) adherence suggesting the pivotal role of diet in preventing the CVD and managing the oxidative stress. Particularly, MD contributes to decreasing the TMAO levels by a microbiota remodeling, mainly by increasing the growth of non-TMA producing species, including *Prevotella* and *Firmicutes* [36]. The remodeling of microbiota, indeed, has been recognized as the main approach for reducing circulating TMAO levels [24]. Besides the use of probiotics and prebiotics, or plant-based diets, the evidence demonstrated that polyphenols may efficiently contribute to this remodeling of the gut microbiota. In particular, polyphenols are effective antimicrobial agents against specific bacterial strains producing TMA, including *Clostridia* and *Bacteroides* [25,37].

According to the available literature, the observed TMAO-reducing effects of Taurisolo^®^ might be attributed to polyphenols. Among the polyphenols contained in grape pomace, RSV has been recently demonstrated to be effective in decreasing TMAO serum levels. Chen et al. [26] conducted an animal-based study demonstrating a TMAO-reducing effect of RSV in ApoE-/- mice. According to the authors, this effect was mainly due to the microbiota remodeling ability of RSV. In particular, they showed that RSV increased the growth of *Lactobacillus* and *Bifidobacterium*. Interestingly, the treatment with antibiotics, abolished the effects of RSV in reducing TMAO levels, confirming the role of RSV in microbiota remodeling [26].

Overall, these studies provide strong evidence for polyphenol-mediated microbiota remodeling, resulting in the reduction of TMAO levels, and suggest that polyphenol-rich diets and/or supplements might be considered as a novel approach for management and/or prevention of CVD and oxidative stress-related diseases.

As previously described, TMAO results from the hepatic oxidation of TMA by FMO3 [7,8] Interestingly, it has been well established that TMAO can also act as an electron acceptor in bacteria [38]. The involvement of the TMAO in redox reactions, allows us to assume a novel interpretation of the TMAO-reducing effect of polyphenols. Polyphenols, indeed, are historically known as the antioxidant molecules par excellence. Additionally, evidence showed that polyphenols are able to exert a strong action as a scavenger of several oxidants. This action is due to phenolic rings with different hydroxyl groups present in the chemical structure. Among these, the hydroxyl group in position 4′ is essential for the RSV scavenger activity [39,40]. At serum level, thus, TMAO and polyphenols may be involved in these redox reactions. In particular, it is plausible to deduce that TMAO may be reduced by polyphenols, acting as electron acceptors and resulting in the generation of TMA. This proposed mechanism might represent a further explication for the reduced TMAO serum levels observed in this study. However, mechanistic studies are needed to confirm the ability of Taurisolo^®^ polyphenols to directly reduce TMAO to TMA.

This hypothesis is corroborated by the results of our bioaccessibility and bioavailability studies.

The profile of the main Taurisolo^®^ polyphenols was described by using an HPLC-DAD analysis, as reported in Table 1. Among these, we considered RSV as the representative polyphenol in Taurisolo^®^, and its levels were monitored in each analysis. In Taurisolo^®^, RSV concentration was 135.7 ± 0.64 µg/g. In order to obtain the best nutraceutical formulation, we tested in vitro the effects of the GI digestion on AR and NAR capsules containing Taurisolo^®^, monitoring the levels of RSV. After GI digestion, in the duodenal phase NAR capsule formulation brought about a 17% loss of RSV content, whereas in AR capsule formulation RSV amounts were less severely affected (3.7%), suggesting that the AR capsule represents an efficient strategy to transport bioactive compounds to the gut and protect them from the effects of GI digestion. 

**Figure 4:**
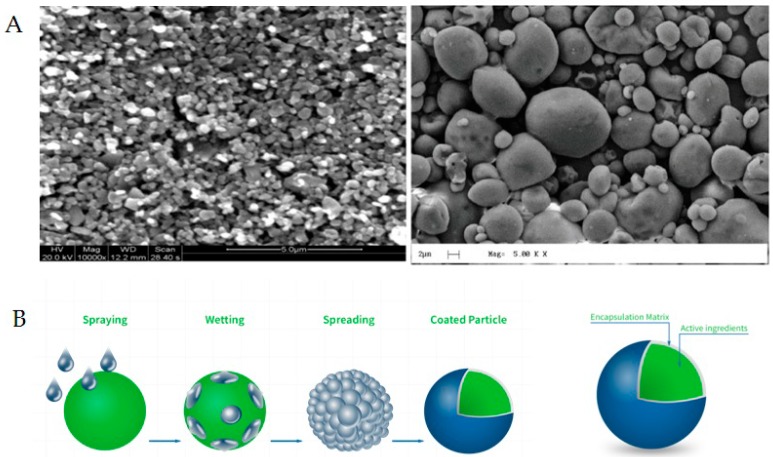
Microencapsulation of grape polyphenolic extract with maltodextrins. (**A**) Representative microphotographs of the Taurisolo^®^ ultrastructure obtained by SEM analysis. (**B**) Graphic representation of the microencapsulation after the spry-drying process. SEM, Scanning Electron Microscopy.

It was well-established that grape polyphenols, and specifically RSV, have a low bioaccessibility and bioavailability [41,42], mainly due to their low water solubility [43]. Several strategies have been developed in order to improve the bioaccessibility and, among these, the use of cyclodextrins as a vector is promising [41]. Taurisolo^®^ is an ethanolic extract microencapsulated in maltodextrins. Although cyclodextrins and maltodextrins are structurally different, the use of the spray-drying process causes a modification in the maltodextrin conformation, passing from linear to circular, incorporating polyphenols and, thus, miming the cyclodextrins, as shown in Figure 4. This microencapsulation process may be responsible for the improvement in RSV bioaccessibility, resulting in enhancement of its water solubility. During the in vitro GI digestion, indeed, after the duodenal stage, all components present in the water solution are considered as potentially absorbed at the intestinal level in vivo. In this context, the levels of RSV after the duodenal stage (considered as RSV bioaccessibility) refer to those detected in water solution after this digestive process.

In addition, the plasma levels of RSV were monitored in each subject, showing that after the administration of the Taurisolo^®^ nutraceutical, they increased both in acute (higher level at 60 min after the administration) and chronic (after the 4-week treatment) cases. These data confirm that, after the oral administration, Taurisolo^®^ polyphenols are absorbed at the intestinal level and reach the bloodstream. In addition, the results obtained from the chronic bioavailability study provide the evidence that after a long-term assumption of Taurisolo^®^, the polyphenols blood levels significantly increased. Our results are in accordance with previous pharmacokinetic studies, which reported that the plasmatic concentration of RSV peaks about 30 min after oral administration [44].

To the best of our knowledge, this study is the first evaluating the TMAO-reducing effects of grape pomace polyphenols in humans. However, the present study is not without limitations. Firstly, we are conscious that the small sample size does not provide strong evidence, although this is a cross-over study. Further limitations are the short duration of the study as well as race and age range chosen, due to the availability of such individuals at the stage of the recruitment. In particular, this study has been conducted on healthy adults, without any symptoms indicative of organ damage. Although there is a well-established relationship between age and CVD risk, we decided to conduct this study on healthy, and relatively young, subjects in order to obtain an overview of the potential of Taurisolo^®^ to control serum levels of a specific prognostic marker indicative of cardiovascular events. This is in line with the major role which must be played by nutraceutical products in general, that is their efficacy in delaying the onset of a pathological state [45]. Moreover, the serum levels of RSV metabolites were not analyzed and in vitro studies were not conducted in order to confirm the potential role of RSV in reducing TMAO to TMA, taking part in a redox reaction. Furthermore, a study of the microbiota in participants before and after treatment, should be useful in demonstrating that Taurisolo^®^ is able to remodel the microbiota, but this was not the main goal of the present study. Conversely, the major strengths of our trial are the originality of the study and the evaluation of the treatment effects in real-world settings. Our results can inform physicians about a novel treatment/intervention as an alternative in the clinical practice. However, this is a pilot study, mainly aimed at evaluating the TMAO-reducing effect of Taurisolo^®^ in healthy subjects. Further investigations, on a higher number of participants, are needed to confirm our results.

## 5. Conclusions

In summary, in the present study, we demonstrated the effect of a novel nutraceutical formulation based on grape pomace polyphenolic extract in reducing the serum levels of TMAO in healthy subjects. The use of AR capsules improves the intestinal bioaccessibility of RSV, resulting in an efficient strategy to transport bioactive compounds to the gut, where they can be absorbed. Although mechanistic studies have not been performed, according to the available literature, we can hypothesize two mechanisms of action by which Taurisolo^®^ may exert its TMAO-reducing effect: the antioxidant activity and the microbiota remodeling, both exerted by polyphenols. Further confirmatory in vitro studies, however, are needed.

## Figures and Tables

**Table 1:** High Performance Liquid Chromatography-diode-array detector (HPLC-DAD) analysis of the main polyphenols contained in Taurisolo^®^. Values are expressed in µg/g Taurisolo^®^ ± standard deviation of three repetitions.

Compound	Mean Value ± SD
Gallic acid	14,634.2 ± 65.5
Syringic acid	5391.8 ± 6.02
Caffeic acid	206.6 ± 0.76
*p*-coumaric acid	278.7 ± 0.66
Ferulic acid	104.8 ± 0.70
Resveratrol	135.7 ± 0.64
Catechin	10,869.8 ± 64.5
Epicatechin	8859.9 ± 7.82
Quercetin	4021.8 ± 7.11
Rutin	284.2 ± 0.70
Procyanidin B1 dimer	628.5 ± 0.59
Procyanidin B2 dimer	4265.2 ± 5.92
Procyanidin B3 dimer	2204.9 ± 6.61
Procyanidin B4 dimer	565.9 ± 0.88
Procyanidin C2 trimer	446.5 ± 0.66

**Table 2:** Pharmacokinetic profile of Taurisolo^®^.

Sample	Time Collection	RSV Content (ng/mL)
Serum	0 min	n.d.
	30 min	5.55 ± 0.02 ^a^
	60 min	49.0 ± 0.55 ^b^
	120 min	3.99 ± 0.04 ^c^
	240 min	2.90 ± 0.06 ^d^
Blood	0 min	n.d.
	30 min	10.6 ± 0.33 ^e^
	60 min	14.2 ± 0.39 ^f^
	120 min	8.13 ± 0.05 ^g^
	240 min	7.98 ± 0.04 ^h^
Serum	4 weeks	7.50 ± 0.04 ^i^

RSV content quantified in serum and whole blood samples of participants in acute and chronic Taurisolo^®^ administration. Values are expressed in ng per mL of serum or whole blood ± standard deviation of three repetitions. ^a,b,c,d,e,f,g,h,i^ Mean values with different superscript letters are significant. Different by Tukey-Kramer multiple comparison test. n.d.: not detected.

**Table 3:** Effects of Taurisolo^®^ on TMAO serum levels in healthy subjects (*n* = 20).

Parameters	Placebo			GPE		
	Initial ^a^	Final ^a^	Δ%	Initial	Final	Δ%
Age (years)	30.0 ± 5.0	-	-	-	-	-
Male sex (No (%))	10 (50.0%)	-	-	-	-	-
White ethnicity (No (%))	20 (100.0 %)	-	-	-	-	-
BMI (kg/m^2^)	20.8 ± 3.6					
TMAO levels (µM) ± SD	1.86 ± 0.35	1.84 ± 0.34 *	−0.54	1.87 ± 0.33 ^#^	0.66 ± 0.44 **^##^	−63.5

Value are expressed in µM ± SD of three replicates. Decrement percentage was shown. Statistic significance is calculated by Student’s *t*-test. ^a^ Initial refers to samples collected at days 8 and 42. Final refers to samples collected at days 35 and 70. * *P* = 0.9297, initial vs. final (placebo); ** *P* < 0.0001, initial vs. final (Taurisolo^®^); ^#^ *P* = 0.9555 placebo vs. Taurisolo^®^ (initial); ^##^ *P* < 0.0001, placebo vs. Taurisolo^®^ (final).

## Supplementary Materials

The Supplementary Materials are available online at http://www.mdpi.com/2072-6643/11/1/139/s1.
