# Supplementary material for: Effects of Grape Pomace Polyphenolic Extract (Taurisolo®) in Reducing TMAO Serum Levels in Humans: Preliminary Results from a Randomized, Placebo-Controlled, Cross-Over Study
Source: Nutrients. 2019 Jan 10;11(1):139. doi: 10.3390/nu11010139 (PMC6356416; doi:10.3390/nu11010139)
Supplement: Supplementary file 1 [file nutrients-11-00139-s001.pdf]

Supplementary materials

Materials and methods

In Vitro Gastrointestinal Digestion Protocol

Two versions of the nutraceutical formulation (acid-resistant (AR) and non acid-resistant (NAR) capsules) were separately mixed to 6 mL of artificial saliva (components: KSCN (20 g/L), KCl (89.6 g/L), Na_2SO_4 (57.0 g/L), NaH_2PO_4 (88.8 g/L), $NaHCO_3$ (84.7 g/L), NaCl (175.3 g/L), urea (25.0 g/L), and 290 mg of α-amylase). The solution pH was adjusted to 6.8 using HCl 0.1 N. The mixture was added to 40 mL of water and homogenized by using a Stomacher 80 Microbiomaster (Seward, Worthing, UK) for 3 min. After this time, 0.5 g of pepsin (14,800 U) dissolved in HCl 0.1 N was added and the solution pH was adjusted to 2.0 using HCl 6 N. The solution was incubated at 37 °C in a Polymax 1040 orbital shaker (250 g) (Heidolph, Schwabach, Germany) for 2 h. Then the pH was adjusted to 6.5 using $NaHCO_3$ 0.5 N and 5 mL of a mixture of pancreatin (8.0 mg/mL) and bile salts (50.0 mg/ mL) (1:1; v/v), dissolved in 20 mL of water, were added and incubated at 37 °C in an orbital shaker (250 g) for 2 h. These latter samples (representing the intestinal phase) were centrifuged at 6,000 g for 10 min and the supernatants were collected and lyophilized. One mg of lyophilized sample was firstly dissolved in 500 µl methanol, vortexed for 1 min, sonicated for 10 min, and then centrifuged (14,000 g, 10 min, 4 °C) to precipitate the salts. The supernatant was collected and 500 µl was added. The sample thus obtained was appropriately diluted and underwent High Performance Liquid Chromatography (HPLC) analysis for the determination of the RSV content.

HPLC/DAD analyses of Taurisolo® polyphenols

The HPLC/DAD analysis was performed using a HPLC system Jasco Extrema LC-4000 system (Jasco Inc., Easton, MD) fitted with an auto sampler, a binary solvent pump, and a diode-array detector (DAD). The separation and quantification were achieved using Synergy Polar-RP C18 column (150x4.6 mm I.D., 4 µm particle size, Phenomenex, Torrance, CA, USA) preceded by a Polar RP security guard cartridge. The column temperature was set at 40 °C. The mobile phase A consisted of 0.1% formic acid in water (v/v) and the mobile phase B consisted of acetonitrile. Injection volume was 20 µL and the flow rate was kept at 1 mL/min for a total run time of 30 min. The gradient program was 0 min 90% (A), 0–17 min 40%(A), 17–22 min 40% (A), 22–28 min 90% (A), and kept at 90% (A) until the end of the run at 30 min. The content of monomeric/oligomeric tannins (catechin, epicatechin, dimers B1, B2, B3, and B4; trimer C2) was assessed according to the method previously described by Silva et al. (2012). Calibration curves of external standards were used for quantification of monitored polyphenols; each curve has been generated by plotting HPLC peak areas against the concentrations (mg/l) (R2 > 0.99).

TMAO quantification

The HPLC system Jasco Extrema LC-4000 system (Jasco Inc., Ithaca, NY) was coupled to an Advion Expression mass spectrometer (Advion Inc., Ithaca, NY) equipped with an ESI source, operating in positive ion mode. The separation of the analytes was performed using a Luna Hilic (5µ particle size, 150x3mm) and security guard colon both supplied by Phenomenex (Torrance, CA, USA) were used. The column temperature was maintained at 60 °C during analysis. Mobile phase A composition: 0.15% formic acid in water containing a final concentration of 10 mM ammonium acetate; mobile phase B composition: methanol. Mobile phases ratio was 80:20 (A:B), run isocratically at a flow rate of 0.35 mL/min for 6 min, with a 5 µl injection volume

Study design, setting, and population

Exclusion criteria were: smoking, obesity (BMI > 30 kg/m2), diabetes, hepatic disease, renal disease, heart disease, family history of chronic diseases, drug therapy or supplement intake for hypercholesterolemia, drug therapy or supplement intake containing grape polyphenols, heavy physical exercise (> 10 h/week), pregnant women, women suspected of being pregnant, women who hoped to become pregnant, breastfeeding, birch pollen allergy, use of vitamin/mineral supplements 2 weeks prior to entry into the study, and donation of blood less than 3 months before the study.

Randomization, concealment, and blinding

A total of 20 eligible patients (10 men and 10 women, 25–35 years of age) were randomly assigned to two sub-groups (each one of 10 subjects). In the case of dropping out before the intervention period, the patient was replaced by the next eligible patient enrolled. The concealed allocation was performed using an internet-based randomization schedule, stratified by study site. An independent investigator, not clinically involved in the trial, generated the random number list. Patients, clinicians, core laboratories, and trial staff (data analysts statisticians) were blind to treatment allocation.

Study outcomes and data collection

Primary endpoints measured were the variations of Trimethylamine N-oxide (TMAO) serum levels. All raw patient ratings were evaluated in a blinded manner at the site of the principal investigator. The decision process was performed according to a consensus document (unpublished standard operating procedure) before unblinding in order to define conclusive primary efficacy data from a clinical perspective.

Safety

Safety was assessed using reports of adverse events provided by study participants as well as laboratory parameters concerning the hepatic and renal function, vital signs (blood pressure, pulse, height, weight, and body mass index), and physical or neurological examinations. These data regarding the safety were collected at days 8, 35, 42, and 70, including adverse events occurring in the first three weeks after cessation of treatments.

Statistics

Methodology

During the trial, it became apparent that dropouts and incomplete diary documentation created missing data that could not be adequately handled by the intended robust comparison.

To deal with the missing data structure, we used a negative binomial, generalized linear mixed effects model (NB GLMM) that not only yields unbiased parameter estimates when missing observations are missing at random (MAR), but also provides reasonably stable results even when the assumption of MAR is violated. Patients who did not provide any diary data (leading to zero evaluable days) were excluded from the MAR-based primary efficacy analysis, according to an "all observed data approach" as proposed by White and colleagues. This approach is statistically efficient without using multiple imputation techniques.

Data retrieved after withdrawal of randomized study treatment were also included in the analysis. Unless otherwise stated, all of the experimental results were expressed as mean ± standard deviation (SD) of at least three replications. Statistical analysis of data was performed by the Student's t-test or two-way ANOVA followed by the Tukey-Kramer multiple comparison test to evaluate significant differences between a pair of means. Cochran's test ($P < 0.1$) were used to assess the statistic heterogeneity. The I2 statistic was also calculated, and I2> 50% was considered as significant heterogeneity across studies. A random-effects model was used if significant heterogeneity was shown among the trials. Otherwise, results were obtained from a fixed-effects model. Percent change in mean and SD values were excluded when extracting SD values for an outcome. SD values were calculated from standard errors, 95% CIs, p-values, or t if they were not available directly.

Previously defined subgroup analyses were performed to examine the possible sources of heterogeneity within these studies and included health status, study design, type of intervention, duration, total polyphenols dose, and Jadad score. Treatment effects were analyzed using PROC MIXED with treatment and period as fixed factors, subject as a random factor and baseline measurements as covariates, and defined as weighted mean differences and 95% CIs calculated for net changes in parameters evaluated. Data that could not meet the criteria of variance homogeneity (Levene's test) and normal distribution (determined by residual plot examination and Shapiro–Wilks test) even after log transformation were analyzed by a nonparametric test (Friedman). The level of significance (α-value) was 95% in all cases($P < 0.05$).

Analysis sets

The full analysis set population included all randomized patients, and patients who did not fail to satisfy a major entry criterion. Patients who did not provide primary efficacy data from efficacy analyses were excluded. The per protocol set consisted of all patients who did not substantially deviate from the protocol; they had two characteristics. Firstly, this group included patients for whom no major protocol violations were detected (for example, poor compliance, errors in treatment assignment). Secondly, they had to have been on treatment for at least 50 days counting from the day of first intake (completion of a certain prespecified minimal exposure to the treatment regimen). Hence, patients who prematurely discontinued the study or treatment were excluded from the per protocol sample.

Patient involvement

No patients were involved in setting the research question or the outcome measures, nor were they involved in developing plans for participant recruitment, or the design and implementation of the study. There are no plans to explicitly involve patients in dissemination. Final results will be sent to all participating sites.

Results

Safety issues

Although no specific toxicity studies have been performed herein, the safety of polyphenol content of grapes has been widely demonstrated by mutagenicity tests, acute/subacute toxicity studies both in mice and human. In particular, the Commission Regulation (EC) No. 258/1997 established 1000 mg as maximum polyphenolic extract daily intake in humans. Accordingly, the Taurisolo® dose adopted for the trial (600 mg/day) was compatible with that regarded as safe in humans. All results from laboratory analysis concerning the hepatic and renal function, indeed,

did not indicate alteration of values after the Taurisolo® treatment period. Other safety assessments, such as vital signs, were all periodically monitored and baseline values did not change substantially during and at the end of the trial.
